# Qualitative and Sensitivity Analysis of the Effect of Electronic Cigarettes on Smoking Cessation

**DOI:** 10.1155/2018/3738584

**Published:** 2018-08-15

**Authors:** Jae Hun Jung, Anna Park, Il Hyo Jung

**Affiliations:** Department of Mathematics, Pusan National University, Busan 46241, Republic of Korea

## Abstract

Recently, the role of the electronic cigarettes (e-cigarettes) in a way to reduce smoking is increasing. E-cigarettes are a device that delivers only the nicotine, and its use is considered less harmful to health compared with tobacco cigarettes. Smokers frequently make use of e-cigarettes as one of the nonsmoking aid devices. In this work, we propose a mathematical model to analyze the effect of e-cigarettes on smoking cessation. The stability and the bifurcation of the model have been discussed. The parameter estimations from the observed data are drawn, and using the parameters, a reasonable smoking model has been designed. Moreover, by considering the sensitivity results depending on the basic reproduction number *R*_0_, the effective strategies that reduce the smokers are investigated. Numerical simulations of the model show that e-cigarettes may somewhat diminish the numbers of smokers, but it does not reduce the number of quitters ultimately.

## 1. Introduction

Smoking is well known as one of the most serious global public health problems. According to the report by the World Health Organization (WHO) [1], smoking is the legalization of a drug that kills many of its users. In other words, smoking leads to disease and disability and harms almost every organ of the body by both active and passive (second-hand) smoking. Moreover, smoking also induces an addiction, so that smokers who want to stop smoking cannot do it. Each year, active smoking is responsible for the death of about 5 million people in the world. In terms of casualties, it is more lethal than tuberculosis, human immunodeficiency virus/acquired immunodeficiency syndrome (HIV/AIDS), and malaria combined. Overall, 600,000 people are estimated to die annually due to the effects of second-hand smoke. Therefore, smoking is a serious health risk.

For that reason, the government encourages enrollment in smoking cessation programs, as well as the use of nicotine patches and nicotine gums so as to reduce the number of smokers. In addition, it establishes no smoking areas. However, since smoking is very difficult to quit, some smokers tend to use electronic cigarettes as a substitute for tobacco cigarettes. E-cigarettes are also considered to be less harmful than tobacco cigarettes [2], as, unlike cigarettes whose smoke contains thousands of harmful substances, such as tar and carbon monoxide, the e-cigarettes contain only nicotine. In fact, from the medical point of view, e-cigarettes have been studied extensively [3, 4]. For example, in [5] and [6], e-cigarettes are described as a valuable product for smoking cessation. However, in [7], the authors suggested that advertising for e-cigarettes must be banned until scientific evidence appears.

On the one hand, from the mathematical modelling point of view, the smoking cessation models have been studied by using mathematical modelling [8–11]. For instance, Sharma and Misra have studied a mathematical model of smoking cessation with media campaigns and bifurcation analysis [12]. Furthermore, Pang et al. have proposed a mathematical model with a saturated incidence rate to explore the effect of controlling smoking [13]. Similarly, Zaman has proposed several mathematical models of an SIR type [14–16].

Although there have been many studies on smoking, to the best of our knowledge, none of previous studies have analyzed the effect of e-cigarettes on smoking cessation using a smoking model. It may be difficult to judge whether e-cigarettes actually help smoking cessation. Therefore, it is worthwhile to study a mathematical model that can identify the characteristics of nonsmokers and quitters, unlike statistical methods and experiments that confirm only the characteristics of simple smokers.

In the present study, we investigate such a smoking model based on the real data from the US Department of Health and Human Services [17]. The aim of this paper is to demonstrate both the addictive nature of smoking and the efficacy of e-cigarettes as an aid in smoking cessation using a mathematical model. Numerical simulations of the model confirm the dynamics of two aspects of the effect of e-cigarettes: the numbers of smokers and nonsmokers.

The rest of the article is composed as follows: In Section 2, a mathematical model to assess the effect of e-cigarettes on smoking cessation is proposed; furthermore, the dynamics of the model, the basic reproduction number, and the stability of equilibria are investigated. In particular, the condition for occurring bifurcation situation is presented. In Section 3, the parameter estimations from real data are provided. The sensitivity analysis and numerical simulation confirm the results that are obtained analytically. Finally, the conclusions are briefly summarized in Section 4.

## 2. Mathematical Model and Analysis

### 2.1. Model

The traditional epidemiologic model of agent, host, vector, and environment is useful for studying the interplay of various influences on patterns of tobacco use in populations. Since the dynamics of smoking cause disease and addiction that is defined when occurring craving, tolerance, withdrawal symptoms, and loss of control, it is very similar to that of an epidemic [18]. For example, a nonsmoker comes into contact with smokers and starts smoking from his influence. Based on epidemic models, therefore, we propose a nonlinear mathematical model to assess the effect of e-cigarettes on smoking cessation.

First, we set up a region with the total population *N* at time *t*. The total population is divided into four subpopulations [19]: potential smokers (*P*) who do not smoke yet but might become smokers in the future; smokers (*S*) who have smoked more than 100 cigarettes in their lifetime and reported smoking “everyday” or “some days” at the time; e-smokers (*E*) who currently use e-cigarettes; and quitters (*Q*) who had smoked more than 100 cigarettes in their lifetime and reported smoking “not at all” at the time. We assume that all of the natural death rates equal to the birth rates is *μ*, and the mortality rate due to the specific diseases that caused by smoking is not considered.

We have considered the effective contact rate, that is, the average number of visits to social gatherings of potential smokers for influential contact of smokers per unit time and the probability of the proportion of becoming smokers after a casual smoking. The constant *β*_1_ is the effective contact rate that potential smokers can become smokers due to peer influence and come in the smokers class at rate *β*_1_*SP*/*N*. The constant *β*_2_ is the effective contact rate, but this case is a relapse to smoking by peer influence. Then quitters can become smokers due to peer influence at rate *β*_2_*SQ*/*N*. In this part, we have considered the effects of peer influence in dynamics of smoking and presented the nonlinear term. Furthermore, we assume that the start smoking rate *β*_1_ is greater than the relapse rate *β*_2_ because the influence of the curiosity of the people who has not smoked is more than people who quit smoking.

**Figure 1:**
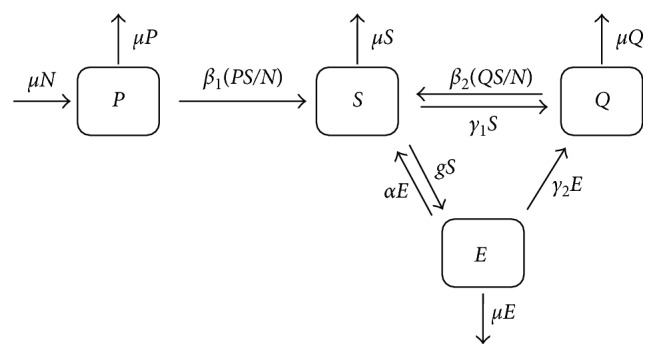
Schematic diagram of system (1).

To quit smoking, however, we consider two kinds of groups: smokers who may quit smoking by their own will at rate *γ*_1_ or smokers who use e-cigarettes at rate *g*. In the latter case, e-smokers who used e-cigarettes are likely to revert to smokers at rate *α*. The constant *γ*_2_ represents the per capita quit rate that stops smoking by use of e-cigarettes. The model parameters are summarized in Table 1. The proposed model is shown in Figure 1 and is described as follows:

(1) $$\begin{array}{c} \frac{dP}{dt}=\mu N-\beta_{1}\frac{PS}{N}-\mu P,\\ \frac{dS}{dt}=\beta_{1}\frac{PS}{N}+\beta_{2}\frac{SQ}{N}+\alpha E-\gamma_{1}S-gS-\mu S,\\ \frac{dE}{dt}=gS-\alpha E-\gamma_{2}E-\mu E,\\ \frac{dQ}{dt}=\gamma_{1}S+\gamma_{2}E-\beta_{2}\frac{SQ}{N}-\mu Q, \end{array}$$

where the initial conditions are given by

(2) $$\begin{array}{c} P\left(0\right)=P_{0}\geq 0,\\ S\left(0\right)=S_{0}\geq 0,\\ E\left(0\right)=E_{0}\geq 0,\\ Q\left(0\right)=Q_{0}\geq 0. \end{array}$$

In system (1), adding all of the equations gives *dN*/*dt*=0 and so the total population is constant, which is equal to *N*. Since system (1) monitors the human population, all the state variables and parameters are nonnegative for all *t* ≥ 0. System (1) shows that the region

(3) $$\begin{array}{c} \Lambda =\left\{\left(P,S,E,Q\right)\in R_{+}^{4}\;\;:\;\;P+S+E+Q=N\right\} \end{array}$$

is positively invariant, which is the region of attraction for the system.

### 2.2. Equilibria

In this section, to find the equilibrium of system (1), it is useful to consider the fractions *P*/*N*=*p*, *S*/*N*=*s*, *E*/*N*=*e*, and *Q*/*N*=*q*, with *p*+*s*+*e*+*q*=1 for convenience of calculation with the reduced form. Then, we can get

(4) $$\begin{array}{c} \frac{dp}{dt}=\mu -\beta_{1}ps-\mu p,\\ \frac{ds}{dt}=\beta_{1}ps+\beta_{2}sq+\alpha e-\gamma_{1}s-gs-\mu s,\\ \frac{de}{dt}=gs-\alpha e-\gamma_{2}e-\mu e,\\ \frac{dq}{dt}=\gamma_{1}s+\gamma_{2}e-\beta_{2}sq-\mu q. \end{array}$$

Substituting *p*=1 − *s* − *e* − *q*, system (4) can be reduced as follows:

(5) $$\begin{array}{c} s^{\prime }=\beta_{1}s\left(1-s-e-q\right)+\beta_{2}sq+\alpha e-\gamma_{1}s-gs-\mu s,\\ e^{\prime }=gs-\alpha e-\gamma_{2}e-\mu e,\\ q^{\prime }=\gamma_{1}s+\gamma_{2}e-\beta_{2}sq-\mu q. \end{array}$$

Here, we investigate the basic reproduction number, *R*_0_, which is defined as the average number of secondary smoking cases produced by one case of smoking that is completely free from e-cigarettes over its smoking period in an entirely potential smoker. If *R*_0_ < 1, then a few smokers will completely replace potential smokers, and the smoking will not spread. If *R*_0_ > 1, then the number of smokers will increase with each generation, and the smoking will spread. To obtain *R*_0_, we have calculated the spectral radius of the next generation matrix used in [21]. Let *x*=(*s*, *e*, *q*)^*T*^, and thus system (5) can be written as

(6) $$\begin{array}{c} \frac{dx_{i}}{dt}=F-V,\;i=1,2,3, \end{array}$$

where

(7) $$\begin{array}{c} F=\left(\begin{array}{c} F_{1}\left(x\right)\\ F_{2}\left(x\right)\\ F_{3}\left(x\right) \end{array}\right)=\left(\begin{array}{c} \beta_{1}s\left(1-s-e-q\right)+\beta_{2}q\\ 0\\ 0 \end{array}\right),\\ V=\left(\begin{array}{c} V_{1}\left(x\right)\\ V_{2}\left(x\right)\\ V_{3}\left(x\right) \end{array}\right)=\left(\begin{array}{c} -\alpha e+\left(\gamma_{1}+g+\mu \right)s\\ -gs+\left(\alpha +\gamma_{2}+\mu \right)e\\ -\gamma_{1}s-\gamma_{2}e+\beta_{2}sq+\mu q \end{array}\right). \end{array}$$

By calculating the Jacobian matrices at *s*=*e*=*q*=0, we find that the derivatives of *ℱ* and *𝒱* lead to the following expressions for *F* and *V*:

(8) $$\begin{array}{c} F=\left(\begin{array}{ccc} \beta_{1} & 0 & 0\\ 0 & 0 & 0\\ 0 & 0 & 0 \end{array}\right),\\ V=\left(\begin{array}{ccc} \gamma_{1}+g+\mu & -\alpha & 0\\ -g & \alpha +\gamma_{2}+\mu & 0\\ -\gamma_{1} & -\gamma_{2} & \mu \end{array}\right). \end{array}$$

Hence, we have simply calculated

(9) $$\begin{array}{c} V^{-1}=\frac{1}{\left(g+\gamma_{1}+\mu \right)\left(\alpha +\gamma_{2}+\mu \right)-\alpha g}\left(\begin{array}{cc} \alpha +\gamma_{2}+\mu & \alpha \\ g & g+\gamma_{1}+\mu \end{array}\right). \end{array}$$

Therefore, we get

(10) $$\begin{array}{c} R_{0}=\rho \left(FV^{-1}\right)=\frac{\beta_{1}\left(\alpha +\gamma_{2}+\mu \right)}{\left(g+\gamma_{1}+\mu \right)\left(\alpha +\gamma_{2}+\mu \right)-\alpha g}, \end{array}$$

where *FV*^−1^ denotes the next generation matrix and *ρ*(*A*) denotes the spectral radius of matrix *A*.

Obviously, system (5) always has a smoking-free equilibrium *e*_0_=(0,0,0) which indicates that smoking does not exist. In addition, system (5) has a smoking-present equilibrium *e*_*∗*_=(*s*^*∗*^, *e*^*∗*^, *q*^*∗*^). Letting the left side equations of system (5) be equal to zero, the smoking-present equilibrium is calculated as follows:

(11) $$\begin{array}{c} e^{*}=\frac{gs^{*}}{\alpha +\gamma_{2}+\mu },\\ q^{*}=\frac{\gamma_{1}s^{*}+\gamma_{2}e^{*}}{\beta_{2}s^{*}+\mu }. \end{array}$$

Substituting the first equation of system (5) into (11), we get the following quadratic equation:

(12) $$\begin{array}{c} a{\left(s^{*}\right)}^{2}+b\left(s^{*}\right)+c=0, \end{array}$$

where

(13) $$\begin{array}{c} a=\beta_{1}\beta_{2}\left(\alpha +\gamma_{2}+g+\mu \right),\\ b=\left(\beta_{1}-\beta_{2}\right)\left\{\left(\alpha +\mu \right)\gamma_{1}+\left(\gamma_{1}+g\right)\gamma_{2}\right\}+\beta_{1}\mu \left(\alpha +\gamma_{2}+\mu +g\right)+\beta_{2}\left\{\left(\alpha +\gamma_{2}+\mu \right)\left(g+\gamma_{1}+\mu \right)-\alpha g\right\}\left(1-R_{0}\right),\\ c=\mu \left\{\left(\alpha +\gamma_{2}+\mu \right)\left(g+\gamma_{1}+\mu \right)-\alpha g\right\}\left(1-R_{0}\right). \end{array}$$

From (12), we get

(14) $$\begin{array}{c} s_{+}^{*}=\frac{-b+\sqrt{b^{2}-4ac}}{2a},\\ s_{-}^{*}=\frac{-b-\sqrt{b^{2}-4ac}}{2a}. \end{array}$$

Here, the coefficient *a* is always positive for all parameter values. But the sign of *b* is changed by parameters, and the sign of *c* depends on *R*_0_. We can consider two cases: (i) if *R*_0_ > 1, then *c* < 0. In this case, each has one negative and one positive solution. It follows that (12) has a unique positive equilibrium. (ii) If *R*_0_ < 1, then *c* > 0. When *b* < 0, (12) has two positive solutions and the number of endemic equilibria may be two.

### 2.3. Stability Analysis

Now, the stability of the equilibria is explored and the following results are obtained.

**Theorem 1 .**

System (5) has the smoking-free equilibrium *e*_0_=(0,0,0) with *p*+*s*+*e*+*q*=1. If *R*_0_ < 1, then *e*_0_ is locally asymptotically stable. Otherwise, if *R*_0_ > 1, then *e*_0_ is unstable.

**Proof**

The Jacobian matrix at *e*_0_ of system (5) is

(15) $$\begin{array}{c} J\left(e_{0}\right)=\left(\begin{array}{ccc} \beta_{1}-\left(g+\gamma_{1}+\mu \right) & \alpha & 0\\ g & -\left(\alpha +\gamma_{2}+\mu \right) & 0\\ \gamma_{1} & \gamma_{2} & -\mu \end{array}\right). \end{array}$$

The eigenvalue from (15) is given by *λ*_1_=−*μ* < 0, and the other eigenvalues are determined by the sub-Jacobian matrix:

(16) $$\begin{array}{c} J_{\text{s}}=\left(\begin{array}{cc} \beta_{1}-\left(g+\gamma_{1}+\mu \right) & \alpha \\ g & -\left(\alpha +\gamma_{2}+\mu \right) \end{array}\right). \end{array}$$

Then, the characteristic equation of *J*_s_ is as follows:

(17) $$\begin{array}{c} \lambda^{2}+A_{1}\lambda +A_{2}=0, \end{array}$$

where

(18) $$\begin{array}{c} A_{1}=\left(\alpha +\gamma_{2}+\mu \right)+\left(g+\gamma_{1}+\mu \right),\\ A_{2}=\left\{\left(\alpha +\gamma_{2}+\mu \right)\left(g+\gamma_{1}+\mu \right)-\alpha g\right\}\left(1-R_{0}\right). \end{array}$$

It follows that *A*_1_ > 0 and *A*_2_ > 0 (i.e., *R*_0_ < 1), and then *e*_0_ is locally asymptotically stable if *R*_0_ < 1, otherwise unstable if *R*_0_ > 1.

**Theorem 2 .**

System (5) has the smoking-present equilibrium *e*_*∗*_=(*s*^*∗*^, *e*^*∗*^, *q*^*∗*^), and it is locally asymptotically stable if *γ*_1_ > *β*_2_*q*^*∗*^ and *β*_1_ > (*α*/*s*^*∗*^).

**Proof**

By setting *ds*/*dt*=0, *de*/*dt*=0, and *dq*/*dt*=0 and dividing the first equation of (5) by *s*^*∗*^, the following can be obtained:

(19) $$\begin{array}{c} \beta_{1}\left(1-s^{*}-e^{*}-q^{*}\right)+\beta_{2}q^{*}+\alpha \frac{e^{*}}{s^{*}}-\left(\gamma_{1}+g+\mu \right)=0. \end{array}$$

From the above equation and system (5), the Jacobian matrix at *e*_*∗*_ of system (5) is

(20) $$\begin{array}{c} J\left(e_{*}\right)=\left(\begin{array}{ccc} -\beta_{1}s^{*}-\alpha \frac{e^{*}}{s^{*}} & \beta_{1}+\frac{\alpha }{s^{*}} & -\beta_{1}+\beta_{2}\\ g & -\left(\alpha +\gamma_{2}+\mu \right) & 0\\ \gamma_{1}-\beta_{2}q^{*} & \gamma_{2} & -\left(\beta_{2}s^{*}+\mu \right) \end{array}\right). \end{array}$$

The characteristic equation of *J*(*e*_*∗*_) is

(21) $$\begin{array}{c} \lambda^{3}+A_{1}\lambda^{2}+A_{2}\lambda +A_{3}=0, \end{array}$$

where the coefficients are given by

(22) $$\begin{array}{c} A_{1}=\beta_{1}s^{*}+\beta_{2}s^{*}+\frac{\alpha e^{*}}{s^{*}}+\alpha +\gamma_{2}+2\mu ,\\ A_{2}=\left(\beta_{2}s^{*}+\mu \right)\left(\beta_{1}s^{*}+\frac{\alpha e^{*}}{s^{*}}+\alpha +\gamma_{2}+\mu \right)+\left(\beta_{1}s^{*}+\frac{\alpha e^{*}}{s^{*}}\right)\left(\alpha +\gamma_{2}+\mu \right)+g\left(\beta_{1}-\frac{\alpha }{s^{*}}\right)+\left(\beta_{1}-\beta_{2}\right)\left(\gamma_{1}-\beta_{2}q^{*}\right),\\ A_{3}=\left(\beta_{1}-\beta_{2}\right)\left\{g\gamma_{2}+\left(\gamma_{1}-\beta_{2}q^{*}\right)\left(\alpha +\gamma_{2}+\mu \right)\right\}+\left(\beta_{2}s^{*}+\mu \right)\left\{\left(\beta_{1}s^{*}+\frac{\alpha e^{*}}{s^{*}}\right)\left(\alpha +\gamma_{2}+\mu \right)+g\left(\beta_{1}-\frac{\alpha }{s^{*}}\right)\right\}. \end{array}$$

And we have

(23) $$\begin{array}{c} A_{1}A_{2}-A_{3}=\left\{\left(\alpha +\gamma_{2}+\mu \right)\left(\beta_{2}s^{*}+\mu \right)+\left(\beta_{1}s^{*}+\frac{\alpha e^{*}}{s^{*}}\right)\right\}\left(\beta_{1}s^{*}+\beta_{2}s^{*}+\frac{\alpha e^{*}}{s^{*}}+\alpha +\gamma_{2}+2\mu \right)+g\left(\alpha +\gamma_{2}+\mu +\beta_{1}s^{*}+\frac{\alpha e^{*}}{s^{*}}\right)\left(\beta_{1}-\frac{\alpha }{s^{*}}\right)+\left(\beta_{2}s^{*}+\mu \right)\left(\beta_{1}s^{*}+\frac{\alpha e^{*}}{s^{*}}\right)+\left(\beta_{2}s^{*}+\beta_{1}s^{*}+\frac{\alpha e^{*}}{s^{*}}+\mu \right)+\left(\beta_{1}-\beta_{2}\right)\left\{\left(\gamma_{1}-\beta_{2}q^{*}\right)\left(\beta_{2}s^{*}+\mu +\beta_{1}s^{*}+\frac{\alpha e^{*}}{s^{*}}\right)-g\gamma_{2}\right\}. \end{array}$$

If *γ*_1_ > *β*_2_*q*^*∗*^ and *β*_1_ > *α*/*s*^*∗*^, then it is apparent that *A*_1_ > 0, *A*_2_ > 0, *A*_3_ > 0, and *A*_1_*A*_2_ − *A*_3_ > 0. Hence, by Routh–Hurwitz criterion, the smoking-present equilibrium *e*_*∗*_ is locally asymptotically stable when the stated condition holds.

### 2.4. Bifurcation

In this section, we provide the dynamics of system (5) at *R*_0_=1, and this transition is called a transcritical (its direction is forward) bifurcation. This means that there is a change in equilibrium behavior at *R*_0_=1, but the equilibrium of smoking population depends on *R*_0_. Besides, we perform a qualitative analysis to investigate the occurrence of backward bifurcations that play an appropriate role in disease control and eradication. In fact, it is well known that in disease transmission modelling, a necessary condition for disease eradication is that the basic reproduction number *R*_0_ must be less than unity [22]. If forward bifurcation occurs when *R*_0_ < 1, then it is a necessary and sufficient condition for smoking extinction. However, if backward bifurcation occurs, an smoking-present equilibrium may also exist for *R*_0_ < 1. In system (5), we calculate a quadratic equation substituting the result of endemic equilibrium *e*_*∗*_ as follows.

Now, applying Theorem 4.1 in [23] to system (5), we obtain a rigorous proof for the forward bifurcation of system (5). We focus on the smoking-free equilibrium *e*_0_ and investigate the occurrence of the transcritical bifurcation with *R*_0_=1 corresponding to

(24) $$\begin{array}{c} \beta_{1}=\beta_{1}^{*}=\frac{\alpha \left(\gamma_{1}+\mu \right)+\left(\gamma_{2}+\mu \right)\left(g+\gamma_{1}+\mu \right)}{\alpha +\gamma_{2}+\mu }. \end{array}$$

First of all, observe the eigenvalues of the matrix:

(25) $$\begin{array}{c} J\left(e_{0};\beta_{1}^{*}\right)=\left(\begin{array}{ccc} \frac{-\alpha g}{\alpha +\gamma_{2}+\mu } & \alpha & 0\\ g & -\left(\alpha +\gamma_{2}+\mu \right) & 0\\ \gamma_{1} & \gamma_{2} & -\mu \end{array}\right), \end{array}$$

which are the roots of the characteristic equation given by

(26) $$\begin{array}{c} \lambda_{1}=0,\\ \lambda_{2}=-\mu ,\\ \lambda_{3}=-\left(\alpha +\gamma_{2}+\mu \right)-\frac{\alpha g}{\alpha +\gamma_{2}+\mu }. \end{array}$$

Since *J*(*e*_0_; *β*_1_^*∗*^) has simple eigenvalues with the zero real part and all eigenvalues have negative real part, system (5) with *R*_0_=1 has a nonhyperbolic equilibrium. Now, a right eigenvalue corresponding to zero eigenvalue, *λ*_1_, denoted by **w**=(*w*_1_, *w*_2_, *w*_3_)^*T*^ is obtained as follows:

(27) $$\begin{array}{c} \left(\begin{array}{ccc} \frac{-\alpha g}{\alpha +\gamma_{2}+\mu } & \alpha & 0\\ g & -\left(\alpha +\gamma_{2}+\mu \right) & 0\\ \gamma_{1} & \gamma_{2} & -\mu \end{array}\right)\left(\begin{array}{c} w_{1}\\ w_{2}\\ w_{3} \end{array}\right)=\left(\begin{array}{c} 0\\ 0\\ 0 \end{array}\right), \end{array}$$

(28) $$\begin{array}{c} -\frac{\alpha g}{\alpha +\gamma_{2}+\mu }w_{1}+\alpha w_{2}=0,\\ gw_{1}-\left(\alpha +\gamma_{2}+\mu \right)w_{2}=0,\\ \gamma_{1}w_{1}+\gamma_{2}w_{3}-\mu w_{3}=0. \end{array}$$

Thus, we get

(29) $$\begin{array}{c} w={\left[1,\frac{g}{\alpha +\gamma_{2}+\mu },\frac{\gamma_{1}\left(\alpha +\gamma_{2}+\mu \right)+\gamma_{2}g}{\mu \left(\alpha +\gamma_{2}+\mu \right)}\right]}^{T}. \end{array}$$

Note that all components of **w** are positive. Furthermore, all components of the left eigenvector **v**=(*v*_1_, *v*_2_, *v*_3_) corresponding to *λ*_1_ which satisfies **v** · **w**=1 given by

(30) $$\begin{array}{c} \left(v_{1}v_{2}v_{3}\right)\left(\begin{array}{ccc} \frac{-\alpha g}{\alpha +\gamma_{2}+\mu } & \alpha & 0\\ g & -\left(\alpha +\gamma_{2}+\mu \right) & 0\\ \gamma_{1} & \gamma_{2} & -\mu \end{array}\right)=\left(\begin{array}{c} 0\\ 0\\ 0 \end{array}\right), \end{array}$$

are positive, and we get the following:

(31) $$\begin{array}{c} -\frac{\alpha g}{\alpha +\gamma_{2}+\mu }v_{1}+gv_{2}+\gamma_{1}v_{3}=0,\\ \alpha v_{1}-\left(\alpha +\gamma_{2}+\mu \right)v_{2}+\gamma_{2}v_{3}=0,\\ -\mu v_{3}=0. \end{array}$$

The left eigenvector **v** is given by

(32) $$\begin{array}{c} v=\left[1,\frac{\alpha }{\alpha +\gamma_{2}+\mu },0\right]. \end{array}$$

The coefficients $\bar{a}$ and $\bar{b}$ defined in Theorem 4.1 in [23] are as follows:

(33) $$\begin{array}{c} \overset{¯}{a}=\overset{3}{\underset{k,i,j=1}{\sum }}v_{k}w_{i}w_{j}\frac{\partial^{2}f_{k}}{\partial x_{i}\partial x_{j}}\left(e_{0},\beta_{1}^{*}\right),\\ \overset{¯}{b}=\overset{3}{\underset{k,i=1}{\sum }}v_{k}w_{i}\frac{\partial^{2}f_{k}}{\partial x_{i}\partial \beta_{1}}\left(e_{0},\beta_{1}^{*}\right). \end{array}$$

Computing them yields the following:

(34) $$\begin{array}{c} \bar{a}=\frac{2\beta_{2}\left\{\left(\gamma_{1}-g\right)\alpha +\gamma_{1}\left(\gamma_{2}+\mu \right)\right\}-\left\{\alpha \left(\gamma_{1}-g\right)+\mu \left(\alpha +g\right)+\left(\gamma_{2}+\mu \right)\left(\gamma_{1}+\mu \right)\right\}}{\mu \left(\alpha +\gamma_{2}+\mu \right)},\\ \bar{b}=\frac{\left(\alpha +\gamma_{2}+\mu \right)\left(2\alpha +\gamma_{2}+\mu \right)}{\alpha g+{\left(\alpha +\gamma_{2}+\mu \right)}^{2}}. \end{array}$$

**Figure 2:**
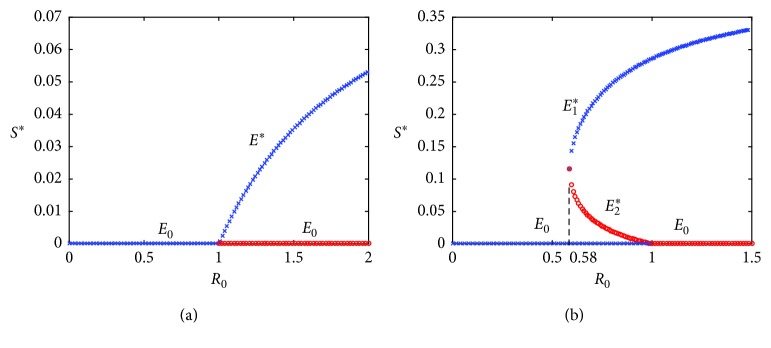
(a) The bifurcation diagram for forward direction (*β*_1_=0.1961, *β*_2_=0.0101); (b) the bifurcation diagram for backward direction (*β*_1_=0.1961, *β*_2_=0.4); cross mark denotes stable equilibrium and dot mark denotes unstable equilibrium.

Therefore, we have the result shown in Figure 2(a).

**Theorem 3 .**

System (5) exhibits transcritical bifurcation at *R*_0_=1 and its direction is forward if

(35) $$\begin{array}{c} \beta_{2}\left\{\left(\gamma_{1}-g\right)\alpha +\gamma_{1}\left(\gamma_{2}+\mu \right)\right\}<\alpha \left(\gamma_{1}-g\right)+\mu \left(\alpha +g\right)+\left(\gamma_{2}+\mu \right)\left(\gamma_{1}+\mu \right). \end{array}$$

On the contrary, we have also checked the occurrence of backward bifurcation in Figure 2(b) because it has a significant impact on prevention strategies and control policies. Indeed, if *γ*_1_ > *g* and *β*_2_ > *β*_1_, then the condition $\bar{a}$ is positive by Theorem 4.1 [23]. Biologically, this means that if a quitting policy is enforced without any preventing measures, then the threshold value that would lead to a state of smoking elimination has to be kept in mind. If the government does not have the policy to reduce smokers, such as smoking cessation campaigns, subsidies for smoking cessation, prevention education, and so on, then the number of smokers will increase because they are not aware of seriousness.

## 3. Numerical Results

### 3.1. Parameter Estimation

In this section, we simulate the parameter estimation for system (1), which allows support for modelling as well as reality. The estimation method is to find the best-fitting curve using the least-squares method in MATLAB. That is, the best-fit sense minimizes the sum of squared residuals that being the difference between the observed value and the fitted value provided by system (1). Here, the mortality rate, *μ*, is estimated by the inverse of life expectancy at birth about total population in the United States [20].

**Figure 3:**
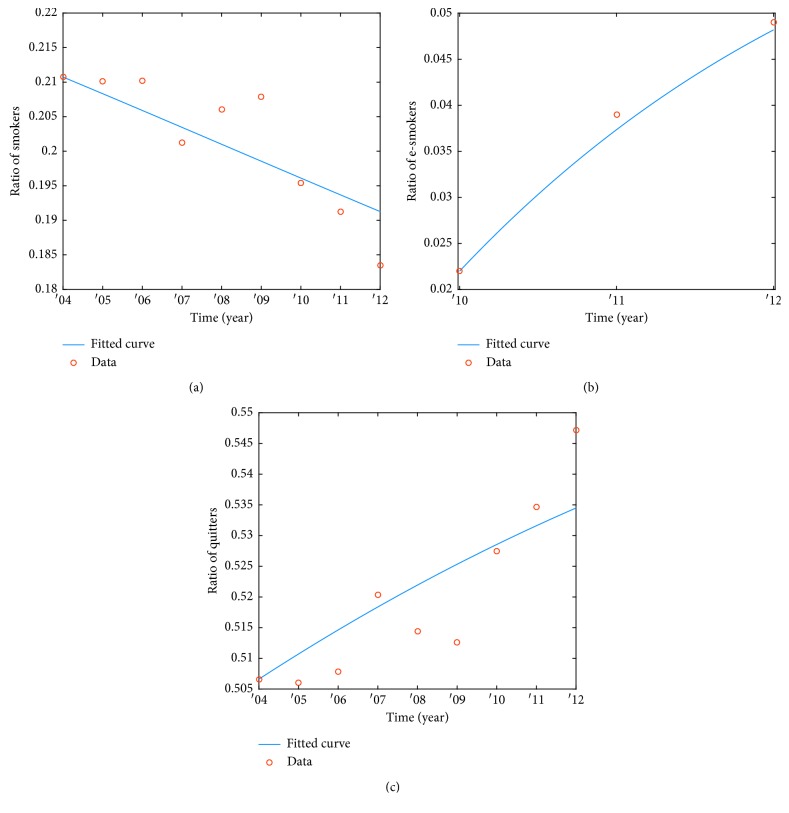
Parameters estimation: (a) the ratio of smokers, (b) the ratio of e-cigarette smokers, and (c) the ratio of quitters.

The real data used in the present study were obtained from the smoking state for 50 years at National Health Interview Survey (NHIS) [17], which reflected the trends in prevalence of current smoking among adults, 18 years of age, and older. First, we had to estimate with data in the absence of e-cigarettes from 2004 to 2012. It is necessary to obtain the transition rate, the recovery rate, and the relapse rate in the original smoking model to verify the efficacy of e-cigarettes later. Therefore, we have obtained the parameter values of *β*_1_, *γ*_1_, and *β*_2_. On the other hand, we could refer to the relationship to cravings [24]. Figures 3(a) and 3(c) show both the number of smokers and quitters referred by NHIS [17], and the data on the number of e-smokers are shown in Figure 3(b), because e-cigarettes were introduced in 2007, but it has been used by a lot of people from 2010 [19]. From these data, we estimate the parameters that are needed to system (1). From the above e-smokers data, we can obtain a ratio of people using e-cigarettes, *g*, and a ratio of people smoking again using e-cigarettes, *α*.

### 3.2. Sensitivity Analysis

In this section, we will analyze sensitivity which decides most effective parameters in progressively reducing the spread of smoking. To do this, we will investigate appropriately all of the possible parameters about the effect of various parameters to reduce basic reproduction number. *R*_0_, close to one. The normalized sensitivity index *𝒮*_*x*_ is the ratio of the corresponding normalized changes for parameters and is defined as follows:

(36) $$\begin{array}{c} S_{x}=\frac{\partial R_{0}/R_{0}}{\partial x/x}=\frac{\partial R_{0}}{\partial x}\frac{x}{R_{0}}. \end{array}$$

Then, we calculate the following:

(37) $$\begin{array}{c} S_{\alpha }=\frac{\alpha g\left(\gamma_{2}+\mu \right)}{M\left(\alpha +\gamma_{2}+\mu \right)}>0,\\ S_{\gamma_{2}}=-\frac{\gamma_{2}\alpha g}{M\left(\alpha +\gamma_{2}+\mu \right)}<0,\\ S_{\gamma_{1}}=-\frac{\gamma_{1}\left(\alpha +\gamma_{2}+\mu \right)}{M}<0,\\ S_{g}=-\frac{g\left(\gamma_{2}+\mu \right)}{M}<0, \end{array}$$

where

(38) $$\begin{array}{c} M=\left(\alpha +\gamma_{2}+\mu \right)\left(g+\gamma_{1}+\mu \right)-\alpha g. \end{array}$$

**Figure 4:**
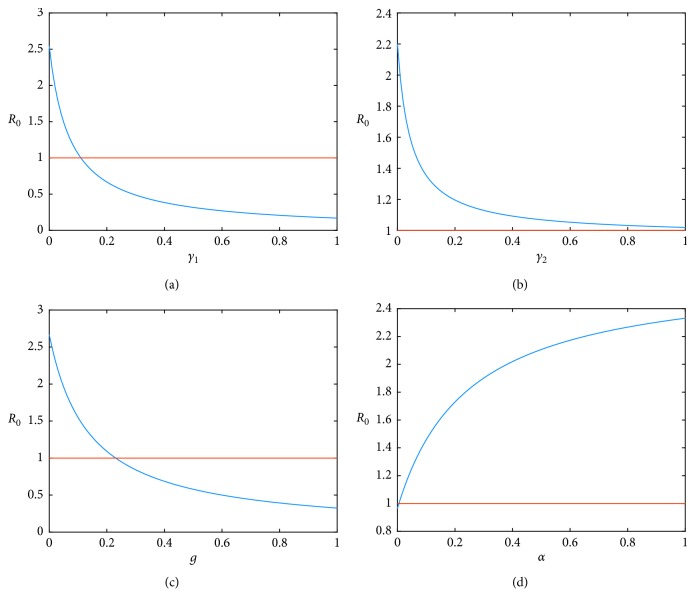
The value of sensitivity indices from the change of each configured parameter value: (a) the rate of quitting smoking by their own will, (b) the rate of quitting smoking by using e-cigarettes, (c) the rate of stopping the use of e-cigarettes, and (d) the rate of using e-cigarettes.

We show that the sign of each sensitivity index determines the relative change of the value of *R*_0_. For instance, if the sensitivity index is positive, then the value of *R*_0_ is an increasing function of the sensitivity index value. On the contrary, the form of the function is decreasing. In Figure 4(d), the sensitivity index of *𝒮*_*α*_ is positive; therefore, as the value of *α* increases, the value of *R*_0_ increases as well. This means that if the government adopts a policy that restricts the use of e-cigarettes, then e-smokers would revert to smoking.

By contrast, in Figures 4(a)–4(c), the sensitivity indices of *𝒮*_*γ*_1__, *𝒮*_*γ*_2__, and *𝒮*_g_ are negative, and these parameters are factors that reduce the number of smokers. As the values of *γ*_1_ and *g* increase, the value of *R*_0_ rapidly decreases. And, these two parameters have a similar tendency to reduce *R*_0_. Biologically, this means that if smokers quit smoking on their own will or switch to e-cigarettes, then the number of smokers will drop substantially. Therefore, these two methods represent the most significant effect on reducing smoking. On the contrary, even if the value of *γ*_2_ increases, the value of *R*_0_ does not fall below one. The impact of *γ*_2_ is weaker than that of the other variables. That is, *γ*_2_ does not have a significant effect on *R*_0_. In addition, the impact of *α* can cause the side effect of e-cigarettes with increasing *R*_0_.Thus, it can be difficult to reduce smokers when using e-cigarettes.

**Figure 5:**
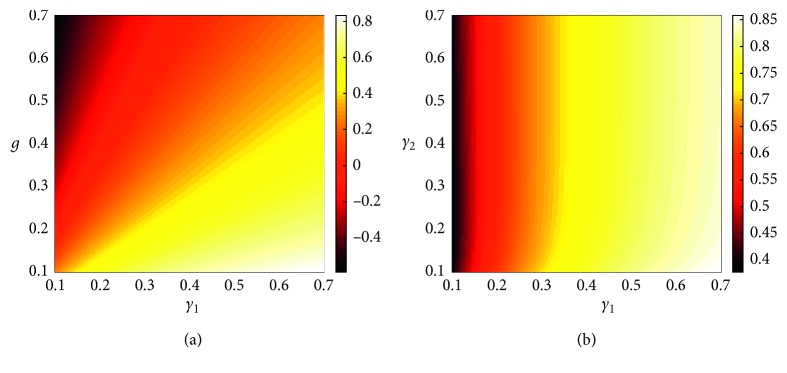
(a) A comparison value of the sensitivity index of *R*_0_ if smoking is stopped by their own will (*γ*_1_) and using e-cigarettes (*g*), that is, *𝒮*_*γ*_1__ − *𝒮*_g_; (b) a comparison value of the sensitivity index of *R*_0_ when smoking is stopped by their own will (*γ*_1_) and when smoking is stopped by using e-cigarettes (*γ*_2_), that is, *𝒮*_*γ*_1__ − *𝒮*_*γ*_2__.

In order to understand how to stop smoking from the perspective of each of the smokers and the quitters, we show the difference of the sensitivity index of *R*_0_ for each parameter regarding the use of e-cigarettes and recovery rate. In Figure 5(a), the value of the sensitivity index of *R*_0_ has both positive and negative signs in relation to two parameters that show a similar pattern. It is effective to both quitting smoking by their own will or using e-cigarettes to reduce smoking. In Figure 5(b), when comparing *γ*_1_ and *γ*_2_, as the value of sensitivity index of *R*_0_ has only positive values, the influence of *γ*_1_ turns out to be stronger. Quitting smoking by using e-cigarettes is less effective than quitting by their own will.

**Figure 6:**
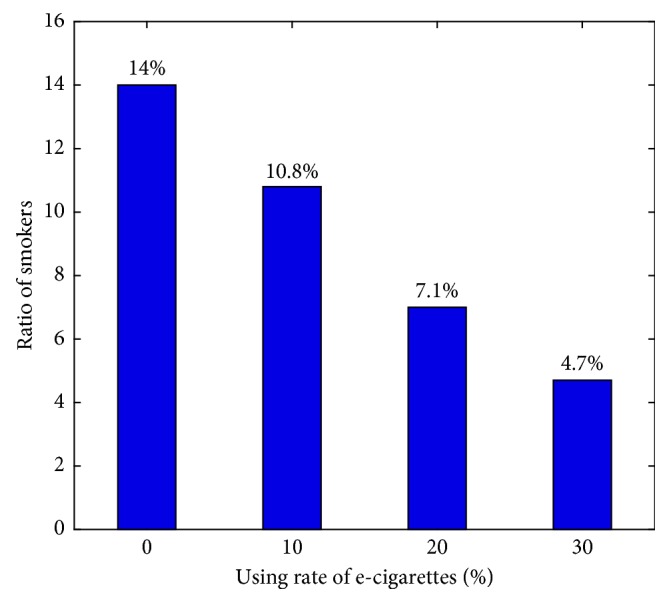
As the using e-cigarettes increases, the ratio of smokers decreases.

Actually, other parameter sets which used the values in Table 1 and Figure 6 are shown to confirm the trend as the rate of using e-cigarettes (*g*) changes. When use of e-cigarettes increases, we can show that the ratio of smokers gradually decreases. In this part, we will consider only the tendency of smokers and determine how e-cigarettes affect smokers.

**Figure 7:**
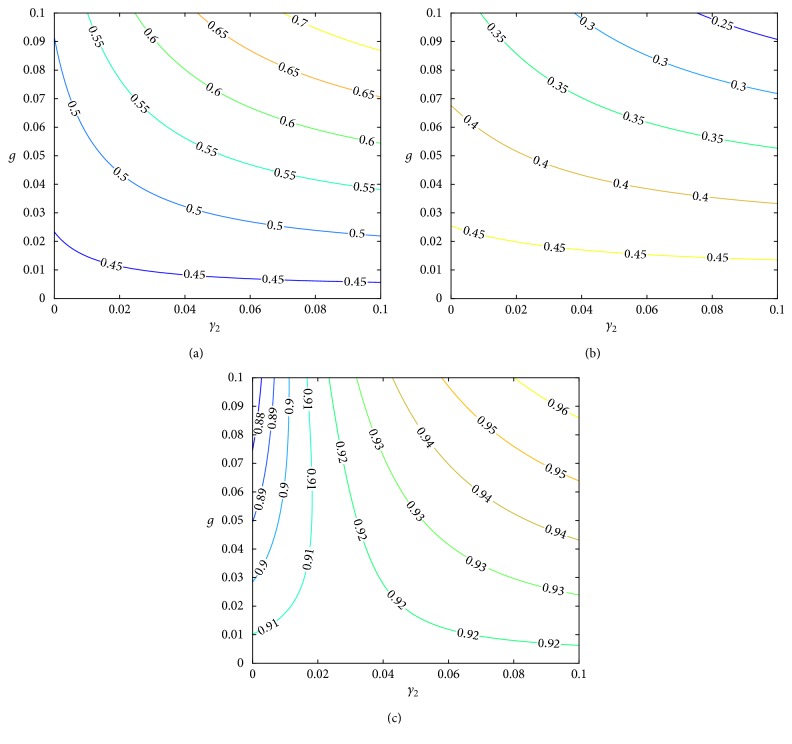
The impact of e-cigarettes on people who do not smoke: (a) the ratio of potential smokers, (b) the ratio of quitters, and (c) the ratio of nonsmokers, that is, potential smokers + quitters.

The effect of e-cigarettes on the ratio of potential smokers, quitters, and nonsmokers can be seen in Figure 7 when used according to the recovery rate of e-cigarettes.  Figure 7(a) shows the high and low recovery rate *γ*_2_ of e-cigarettes; the ratio of potential smokers increased more rapidly when the recovery rate was higher than when the recovery rate was lower. On the contrary, in Figure 7(b), according to the rate of use of e-cigarettes, the ratio of quitters decreased when the recovery rate was higher than when the recovery rate was lower. That is, the ratio of potential smokers and quitters may have contradicting results depending on the recovery rate.

Biologically, we can think of the rate of recovery as information for e-cigarette efficacy because there can be a variety of results in places where there is an advertisement by the government, publicity of e-cigarette companies, information by a medical institution, and so on. Therefore, when the effect is large, the usage is increased, that is, the ratio of people using e-cigarettes is increased and the ratio of people who smoke is decreased; smokers are less encouraged smoking to potential smokers, which increases potential smokers. However, although e-cigarettes are used to stop smoking, the number of cases returning to smoking without being able to overcome the temptation to smoke is increased. That is why quitters are increased. Therefore, when considering two aspects, the number of smokers decreases and the number of the combined potential smokers and quitters increases. Using e-cigarettes may help smokers quit smoking, but a lot of e-cigarettes do not help to quit smoking easily, and quitting e-cigarettes may itself be difficult.

Figure 7(c) represents the sum of Figures 7(a) and 7(b) as a ratio of nonsmokers. From this point of view, we can see how the ratio of nonsmokers varies depending on the rate of use of e-cigarettes. It can be seen that the ratio of nonsmokers decreases evenly as the ratio of using e-cigarettes increases when the recovery rate is remarkably lower. However, when the recovery rate is higher than a certain level, the ratio of nonsmokers increases as the use of e-cigarettes increases.

## 4. Conclusion

In this paper, our main interest was to analyze the effect of e-cigarettes on smoking cessation. We established a mathematical model for smoking cessation with e-cigarettes and presented the theoretical study for the effect of e-cigarettes. The observed real data of the ratio of potential smokers, smokers, the use of e-cigarettes, and quitters are obtained, and the parameters are estimated by the least-square method.

As our results demonstrate, the proposed model can support the reason for modeling because it follows current trends and there are few errors in this estimation. In epidemiology, one of the most important parts, basic reproduction number, *R*_0_ is obtained by the next generation method. From this, we observed that when *R*_0_ < 1, the smoking-free equilibrium *e*_0_ is locally asymptotically stable, whereas when *R*_0_ > 1, the smoking-present equilibrium *e*_*∗*_ is locally asymptotically stable. This means that, if we control the smoking for public health, then the number of smokers in a region can persist if the value of *R*_0_ is greater than unity, or else its users diminish.

The bifurcation analysis presents forward bifurcation at *R*_0_=1, namely, smoking control may be carried out by reducing *β*_1_ below the transcritical value *β*_1_^*∗*^ in order to make *R*_0_ < 1. On the contrary, we should check the backward bifurcation scenario in other conditions where *γ*_1_ > *g* and *β*_2_ > *β*_1_, because smoking has two biggest factors, which are the risk of peer influence and relapse. If smokers stop smoking on their own, rather than using e-cigarettes, there may be a possibility that they will again start smoking if they are at places where there are a lot of smokers. But in the opposite case, if they are in a place where there are not many smokers, they may possibly stop smoking gradually without using e-cigarettes. For example, if smokers go back to smoking when they stop smoking on their own will, they think that the smokers will be able to quit smoking again by themselves. The sensitivity analysis about each parameter can show the effect for *R*_0_, which checks the sign of the sensitivity index. In order to show the effect of e-cigarettes, especially, we consider the ratio of smokers using e-cigarettes and the ratio of nonsmokers by the parameters *γ*_2_ and *g*. From the perspective of the reducing number of smokers, using e-cigarettes can reduce the ratio of smokers, regardless of the recovery rate *γ*_2_. On the contrary, in regard to the increasing number of nonsmokers, potential smokers and quitters tend to be the opposite when the use of e-cigarettes increases.

In conclusion, we verified the efficacy of e-cigarettes from a theoretical standpoint by constructing a mathematical model for various scenarios that could not be captured by statistical or experimental methods. Our results revealed that meaningful differences in the level of use and recovery rate of e-cigarettes change within a group, and we could explain its utility. We consider that this fact may be due to addiction to e-cigarettes. Therefore, the proper use of e-cigarettes can reduce the craving for tobacco cigarettes and may be an effective way to postpone smoking. However, smokers and e-cigarette smokers should never to be overlooked for a possibility of addiction and relapse. Using e-cigarettes may somewhat diminish the numbers of smokers, but it does not increase the number of quitters significantly. Therefore, people who know the seriousness of smoking should prevent the people around from trying to smoke and raise awareness about the adverse impacts of smoking on health, which are pivotal for prevention of smoking.

## Figures and Tables

**Table 1:** Parameter description.

Par.	Description	Value (year^−1^)	Reference
*μ*	Mortality rate	1/79.8	[20]
*β*_1_	Rate at which someone is transitioned to smoking	0.1961	Estimated in [16]
*β*_2_	Rate of relapse	0.0101	Estimated in [16]
*γ*_1_	Treatment rate of people quitting to smoke by their own will	0.0772	Estimated in [16]
*γ*_2_	Treatment rate of people who used e-cigarettes	0.1008	Estimated
*α*	Rates at which people stop using e-cigarettes	0.0822	Estimated
*g*	Rates at which people begin using e-cigarettes	0.1245	Estimated
